# The mediating effect of social support on the association between socioeconomic status and self-reported oral health status among the migrant elderly following children in Weifang, China: a cross-sectional study

**DOI:** 10.1186/s12903-022-02649-6

**Published:** 2022-12-18

**Authors:** Mingli Pang, Jieru Wang, Tingting Tian, Jinfeng Zhao, Xiaoxu Jiang, Hexian Li, Fanlei Kong

**Affiliations:** 1 Centre for Health Management and Policy Research, School of Public Health, Cheeloo College of Medicine, Shandong University, Jinan, 250012 China; 2 NHC Key Lab of Health Economics and Policy Research, Shandong University, Jinan, 250012 China

**Keywords:** Self-reported oral health status, Social support, Socioeconomic status, Migrant elderly following children, Structural equation modeling

## Abstract

**Background:**

Driven by population aging and the rapid urbanization in China, many migrant elderly following children (MEFC) moved to big cities to care for their grandchildren. The purpose of this study is to clarify the mediating effect of social support on the relationship between socioeconomic status (SES) and self-reported oral health status among the MEFC in Weifang, China.

**Methods:**

Multistage cluster random sampling was used to select the participants and finally 613 MEFC were included in the survey. The Social Support Rating Scale (SSRS) and the Chinese version of the Geriatric Oral Health Assessment Index (GOHAI) scale were used for data collection. Descriptive analysis, Rao-Scott test, t-test and structural equation modeling (SEM) were conducted in this study.

**Results:**

Mean score of GOHAI of the MEFC was 54.95 ± 6.47. The SES of MEFC exerted positive direct effect both on social support (standardized coefficient = 0.15) and self-reported oral health status (standardized coefficient = 0.22); social support exerted positive direct effect on self-reported oral health status (standardized coefficient = 0.17). Social support partially mediated the association between SES and self-reported oral health status [95% confidence interval (CI) 0.003–0.064, *P* < 0.05], and the mediating effect of social support accounted for 12.0% of the total effect.

**Conclusions:**

Higher GOHAI score of MEFC indicated their better self-reported oral health status. MEFCs’ SES could exert positive effect both on social support and self-reported oral health status, while the mediating effect of social support between SES and self-reported oral health status of MEFC was established.

## Background

Oral health is a key indicator of overall health, well-being and quality of life, it had proved the relationship between oral and general health [1]. Oral diseases posed a major health burden for many countries and affected people throughout their lifetime, causing pain, discomfort, disfigurement and even death, while oral diseases were caused by many modifiable risk factors and it could largely preventable [2]. Poor oral health among old-age people was an important public health issue and a growing burden to countries worldwide [3].As a group of old-age people, the migrant elderly following children (MEFC) with the dual vulnerable attributes of migration and elderly population [4], MEFC with poor oral health not only suffered from physical discomfort [5], but also led to social isolation [6]. Consequently, the oral health status of MEFC is a significant issue worthy of further researches.

Socioeconomic status (SES) mainly included factors such as income, education, and occupation [7], which was the most overwhelmingly significant risk factor for health and well-being [8], people with low SES were associated with a greater severity of caries [9, 10]. A study in China showed that high income was the protective factor for oral health in older adults [11] and the older adults in South Korea who were from urban area and in good economic conditions had better self-rated oral health status [12]. Positive association between educational level and oral health had been confirmed among older adults [13, 14]. A study indicated that the prevalence of the periodontal disease was found to be significantly higher among the production line workers than administration workers [15]. Studies among preschool children [16, 17] and adolescence [18] also showed that SES was positively associated with oral health. Another study showed that the impact of SES at baseline on oral health–related quality of life at follow-up would be mediated by individual factors, such as social support [19].

Social support was often defined as material and moral support and the exchange of material and moral resources between individuals [20, 21]. Previous studies had showed that social support was associated with periodontal disease in elderly Brazilians [22], individuals were more likely to seek dental care when there was a higher level of social support [23]. Several studies showed social support played an important role in improving oral health outcomes for immigrants [24–26]. A larger number of studies had shown that individuals with lower levels of social support often had poorer oral health [27, 28].

Many existed researches had explored the relationship between SES and social support, the results mainly showed that there was a positive relationship between SES and social support [29, 30] and low SES individuals were unable to mobilize social support when needed [31, 32]. Previous studies had found that household monthly income was associated with social support [33] and financial social support was more needed when the SES (especially income) was lower [34]. Previous study had found that the old adults with higher education level had better social support, and the protective qualities of social support were stronger among the individuals who were less educated [35, 36]. A study among teachers, soldier and local health care workers found that local healthcare workers had the lowest social support while soldier had the highest social support [37].

To conclude, previous researches had explored the relationship between SES and oral health, social support and oral health, SES and social support even the mediating effect of social support (social support as a part of individual factors) between SES and oral health, yet no study had clarified the relationship between the SES, social support (social support was separately as a mediating variable) and self-reported oral health status among the MEFC. Thus, this study aimed to investigate the mediating effect of social support on the relationship between SES and self-reported oral health status among the MEFC in Weifang, China. According to the research purpose, four hypotheses were proposed as follow: H1. SES positively predicted self-reported oral health status. H2. Social support positively affected self-reported oral health status. H3. SES positively predicted social support. H4. SES affected oral health through the mediating effect of social support.

## Methods

### Data collection

A total of 613 MEFC was selected in Weifang City, Shandong Province, China in August 2021. Weifang City is located in the eastern part of China. Up to November 30, 2020, the total household population of the city was 9.19 million and the urbanization rate of the household population was 53.76% [38].

Multistage cluster random sampling was used to select the participants. In the first stage, four districts, Gaoxin district, Kuiwen district, Weicheng district, and Fangzi district, were selected as the primary sampling units (PSUs) based on consideration of the economic development and geographical location of Weifang city. In the second stage, four sub-districts from each PSUs were selected as secondary sampling units (SSUs). In the third stage, four communities were selected from each of the SSUs. The migrant population aged 60 years or older in the selected communities who came to live in Weifang with their children constituted the entire sample for this study. The inclusion criteria for participants were: (1) 60 years old and above; (2) non-local people; (3) come to Weifang for 3 months and above; (4) clear awareness and cognition.

The formula used to calculate the sample size is as follows:

$$n=deff\frac{{u}^{2}p(1-p)}{{\varepsilon }^{2}}$$

In the formula above, the design efficiency $deff$ = 2.5, the level of confidence µ = 1.96, the margin of error ε = 10%, the prevalence of caries in the age group above 65 years old in the third national epidemiological survey of oral health status was *p* = 86.0%, and the non-response rate was 10% [39], so a minimum of 464 participants would be needed for the study.

After completing the offline training on research background, questionnaire content, and social survey techniques, a total of 25 university students were assigned as research investigators for this study, the investigators conducted face-to-face interviews with each participant for approximately 20 min and the total investigation lasted 1 week. Initially, 616 MEFCs were selected and interviewed. However, three participants were excluded as they answered their questionnaires incorrectly or incompletely. Ultimately, a total of 613 participants were included in the database.

### Measurement

The main information collected from the survey included: (1) sociodemographic characteristics: gender, age, *hukou* (a household registration system used in China), marital status; (2) SES: education, job before retire and monthly household income; (3) social support: Social Support Rating Scale (SSRS) was used to assess the social support of MEFC; (4) self-reported oral health status: Geriatric Oral Health Assessment Index (GOHAI) scale was used to assess the self-reported oral health status of MEFC.

#### Socio-demographic characteristics

According to the previous study [40, 41], socio-demographic information included the following four parts: gender (male, female), age, *hukou* (rural, urban), marital status (married or unmarried, divorced, widowed).

#### SES

Social stratification refers to the range of social classes that result from variations in socioeconomic status [42], which are usually measured by the social factors like wealth, income, education, family background, and power [43]. In the field of sociology, Karl Marx and Max Weber were two famous sociologists on social stratification/class theory, as well as in China. Karl Marx created his social stratification theory based on the unequal access to the means of production which created a distinction between the bourgeoisie and proletariat [44, 45], while Max Weber formed a three-component theory of stratification in which social difference was determined by class, status and power [46, 47]. Based on Marx and Weber’s theory, some Chinese scholars had put forward different research conclusions from different angles on social stratification of the Chinese population, such as Xueyi Lu’s "gentrificating modern society theory" which believed that the trend of gentrification of the modern social structure is emerging in China; Qiang Li’s "fragmentation theory" which emphasized the diversity of the social differentiation while the hierarchical structure is difficult to form in China [48]. Combining the above Western and Chinese scholars’ stratification theory which highlighted the important role of income and occupation, this study furtherly added education to assess the MEFC’s SES; that is, SES was measured by three variables in this study: monthly household income (≤ 240 Renminbi (RMB), 241–3000 RMB, > 3000 RMB), job before retire (agriculture, forestry, animal husbandry and fishery, others), and education (primary school and below, junior high school, high school and above).

#### SSRS

SSRS was developed by Xiao [49], included 10 items and three dimensions: objective social support, subjective social support and social support utilization. The scale score varies from 12 to 66 and were classified into three levels: high (45–66), medium (23–44), low (≤ 22), the higher the score, the higher the level of social support. Existed researches showed SSRS had good reliability and validity in Chinese population [50].

#### GOHAI

GOHAI scale is a general oral health assessment index, proposed in 1990 by Atchison and Dolan [51]. The main application of the scale is for the elderly and had been translated into Chinese [52, 53], French [54], Urdu [55] and other languages [56]. GOHAI contains three dimensions: physiological function, psychosocial function, pain and discomfort, and had good reliability and validity in Chinese population [52]. The total GOHAI score was used to classify the oral health of the elderly into three levels: high (57–60), medium (51–56), and low (≤ 50) [51, 52]; the higher score of total GOHAI scale indicating the better of self-reported oral health status.

### Analytical approach

Descriptive statistics was used to describe the socio-demographic characteristics of the participants. The Chi-square test was employed to clarify the *hukou* differences in the socio-demographic characteristics (gender, age and marital status), and t-test was used to assess the *hukou* differences in the self-reported oral health status and social support indicators respectively. *P*-values of less than 0.05 were regarded as statistically significant. All the analyses above were performed by using SPSS version 24.0 (SPSS Inc., Chicago, IL, USA).

SEM was conducted to examine the relationship between SES, social support and self-reported oral health status of the MEFC by using SPSS Amos 24.0 (IBM Corp., Armonk, NY, USA). The model in the SEM consists of two kinds of variables: exogenous variable and endogenous variable. In this study, the endogenous variables were social support and self-reported oral health status, while the exogenous variable was SES.

Assessment of the model fitness calculates how the proposed model might be consistent with the empirical data. Maximum-likelihood estimation was used to estimate the best-fitting model in this study. The Chi-square value is called CMIN in Amos. CMIN/DF, GFI, AGFI, CFI, NFI, IFI, RMSEA would be adopted as the fitness indices in the current study. The models were regarded to be a good fit when *P* > 0.05; CMIN/DF range 1 to 3; GFI > 0.90; AGFI > 0.90; NFI > 0.90; IFI > 0.90; CFI > 0.90 and RMSEA < 0.05 [57].

Bootstrap tests (sampling process was repeated 1000 times) were conducted to determine the total, indirect and direct effect of the model in SEM [58]. The indirect effect was regarded as statistically significant if the 95% confidence interval (CI) excluded zero and thus the mediating effect did exist in this way.

### Ethical considerations

The survey and data use had obtained the informed consent of all participants. The research program of this was reviewed and approved by the ethical committee of Shandong University (No. 20180225). All methods were carried out in accordance with relevant guidelines and regulations.

## Results

### Sample characteristics

Table 1 shows the basic information of socio-demographic characteristics, SES, social support and self-reported oral health status of MEFC. It was illustrated that 85.6% of the MEFC were from rural areas, while 14.4% from urban areas; most MEFC were female (73.1%), aged 60–65(55.8%), married (87.9%). As for SES, majority of MEFC had an education level of primary school and below (56.4%), job before retire were farming, forestry, livestock and fishing (75.2%), monthly household income were 241–3000 RMB (47.1%). With respect to social support, the mean score of social support was 38.88 ± 6.63 among the MEFC in Weifang City, China. As for the self-reported oral health status, the mean score of GOHAI was 54.95 ± 6.47 for the MEFC in Weifang City, China.

**Table 1:** Descriptive characteristic of participants by *hukou*

Variable	*n* (%)/M(S.D.)	Rural	Urban	χ^2^/t	*P*
		*n* (%)/M(S.D.)	*n* (%)/M(S.D.)		
Observations	613 (100)	525 (85.6)	88 (14.4)		
*Gender*				17.949^a^	0.001
Male	165 (26.9)	125 (23.8)	40 (45.5)		
Female	448 (73.1)	400 (76.2)	48 (54.5)		
*Age*				11.684^a^	0.009
60–65	342 (55.8)	304 (57.9)	38 (43.2)		
66–70	171 (27.9)	143 (27.2)	28 (31.8)		
71–80	80 (13.1)	65 (12.4)	15 (17.0)		
> 80	20 (3.2)	13 (2.5)	7 (8.0)		
*Marital status*				0.860^a^	0.354
Married	539 (87.9)	459 (87.4)	80 (90.9)		
Unmarried/Divorced/Widowed	74 (12.1)	66 (12.6)	8 (9.1)		
*Education*				63.457^a^	0.001
Primary school and below	346 (56.4)	322 (61.3)	24 (27.3)		
Junior high school	158 (25.8)	135 (25.7)	23 (26.1)		
High school and above	109 (17.8)	68 (13.0)	41 (46.6)		
*Job before retire*				208.864^a^	0.001
Farming, Forestry, Livestock and Fishing	461 (75.2)	449 (85.5)	12 (13.6)		
Others	152 (24.8)	76 (14.5)	76 (86.4)		
*Monthly household income*				176.751^a^	0.001
≤ 240 RMB	270 (27.7)	168 (32.0)	2 (2.3)		
241–3000 RMB	289 (47.1)	275 (52.4)	14 (15.9)		
> 3000 RMB	154 (25.1)	82 (15.6)	72 (81.8)		
*Social support*	38.88 (6.63)				
Utilization	6.94 (2.26)	6.86 (2.23)	7.43 (2.37)	− 2.21^b^	0.027
Subjective	23.47 (4.29)	23.45 (4.76)	23.58 (5.00)	− 0.229^b^	0.819
Objective	8.47 (1.64)	8.44 (1.61)	8.70 (1.78)	− 1.425^b^	0.155
*Self-reported oral health status*	54.95 (6.47)				
Physiological function	17.35 (3.44)	17.21 (3.49)	18.18 (3.03)	− 2.730^b^	0.007
Psychosocial function	24.10 (2.06)	24.03 (2.15)	24.50 (1.35)	− 2.725^b^	0.007
Pain and discomfort	13.50 (2.12)	13.41 (2.14)	14.03 (1.86)	− 2.841^b^	0.005

a = Rao-Scott test; b = t-test; *S.D.* Standard deviation

Concerning the rural and urban differences, as shown in Table 1, statistically significant differences were found between *hukou* and gender (*P* < 0.001), age (*P* = 0.009), education (*P* < 0.001), job before retire (*P* < 0.001), monthly household income (*P* < 0.001), and social support utilization dimensions (*P* = 0.027). It was noted that statistically significant difference was also found between *hukou* and the three dimensions of GOHAI (physiological function (*P* = 0.007), psychosocial function (*P* = 0.007), pain and discomfort (*P* = 0.005).

### Model fitness indices

Table 2 shows the model fitness indices of the structural equation model. The estimated value of model fitness for the model were: CMIN/DF = 1.675 in the range of 1–3, GFI = 0.985 > 0.90, AGFI = 0.973 > 0.90, NFI = 0.967 > 0.90, IFI = 0.986 > 0.90, CFI = 0.986 > 0.90 and RMSEA = 0.033 < 0.05, indicating that the theoretical model fit the empirical data very well.

**Table 2:** Structural equation model fitness index

Indexes	CMIN/DF	GFI	AGFI	NFI	IFI	CFI	RMSEA	*P*
Cut-off value	1–3	≥ 0.90	≥ 0.90	≥ 0.90	≥ 0.90	≥ 0.90	≤ 0.05	> 0.05
Observations	1.675	0.985	0.973	0.967	0.986	0.986	0.033	0.020

*CMIN* Chi square, *DF* degree of freedom, *GFI* Goodness of Fit Index, *AGFI* Adjusted Goodness of Fit Index, *NFI* Normed Fit Index, *IFI* Incremental Fit Index, *CFI* Comparative Fitness Index, *RMSEA* Root-mean square error of approximation

### The standardized total, direct, and indirect effects on self-reported oral health status with social support as mediators

**Fig. 1:**
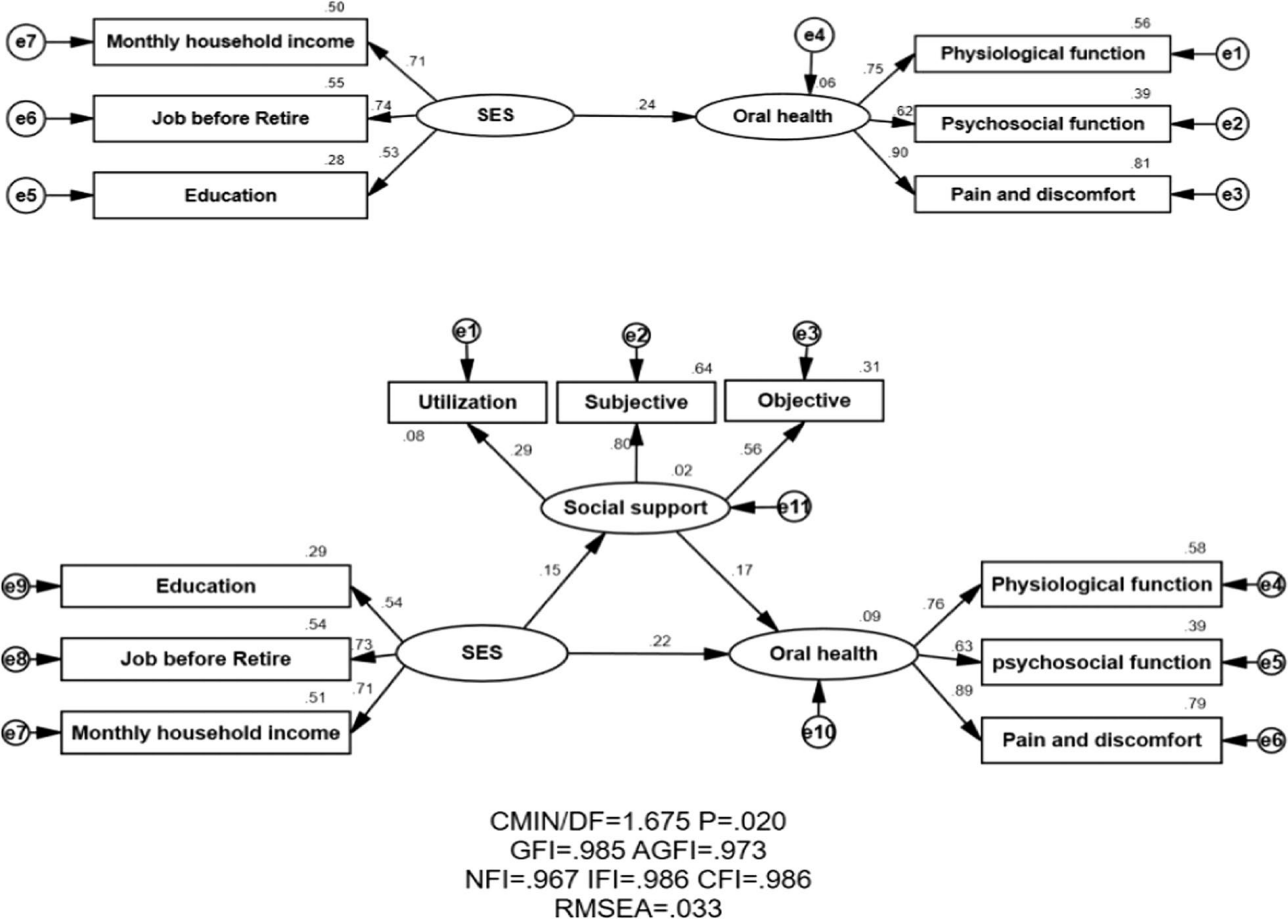
The mediation model of the association between SES and self-reported oral health status through social support

Specifically, bootstrap test suggested that after adjusting for covariates, Fig. 1 and Table 3 showed that the total effect of SES on self-reported oral health status was 0.24 (95% CI 0.137–0.323, *P* < 0.005). The direct effect of SES on self-reported oral health status was 0.22 (95% CI 0.105–0.317, *P* < 0.001). The indirect mediating effect via social support was 0.03 (95% CI 0.003–0.064, *P* < 0.001). The effects above were all statistically significant since the 95% CI excluded zero, implying he association between SES and self-reported oral health status was partially mediated by social support; of which the indirect effect accounted for 12.0% of the total effect.

**Table 3:** The standardized total, direct, and indirect effects

Model pathways	Standardized effect value	S.D	95% C.I	Mediating effect (%)
Total effect SES → self-reported oral health status	0.25*	0.053	(0.115, 0.336)	100
Direct effect SES → self-reported oral health status	0.22*	0.053	(0.105, 0.317)	88
Indirect effect SES → social support → self-reported oral health status	0.03*	0.015	(0.003, 0.064)	12
SES → social support	0.15**			
Social support → self-reported oral health status	0.17**			

*SES* socioeconomic status, *S.D.* Standard Deviation, *CI* Confidence Interval; *P-value < 0.001; **P-value < 0.05. The estimated value of model fitness for the model were: CMIN/DF = 1.675 in the range of 1–3, GFI = 0.985 > 0.90, AGFI = 0.973 > 0.90, NFI = 0.967 > 0.90, IFI = 0.986 > 0.90, CFI = 0.986 > 0.90 and RMSEA = 0.033 < 0.05

## Discussion

### Self-reported oral health status of MEFC in Weifang, China

The mean score of the GOHAI was 54.95 ± 6.47 among the MEFC in Weifang, China, while statistically significant difference of the mean score of the GOHAI between the urban and rural MEFCs were found. Comparing with the GOHAI score of the elderly in Minhang District, Shanghai, China (39.68 ± 7.65) [59], the self-reported oral health status of MEFC in Weifang was higher. This may be due to the average age of MEFC included in our database was 66.29 years old, while the average age in that study was 68.33 years old. Generally speaking, the self-reported oral health status of the younger elderly tended to be better than the older elderly. Moreover, most of the elderly who migrated with their children to take care of grandchildren tend to be in better global health status compared to other elderly since the baby caring needs more physical power and energy. Another study found that the mean score of GOHAI among the middle-aged and older adults in Guangxi Zhuang Autonomous Region (provincial level), China was 56.00 ± 7.00 [60], which was similar to the findings of this study. Moreover, the differences between urban and rural areas on self-reported oral health status was similar to a study conducted among the elderly in Sichuan Province, China [61].

### The mediating effect of social support on the relationship between SES and self-reported oral health status of MEFC in Weifang, China

#### The relationship between SES and self-reported oral health status

It was found that SES could exert its positively direct effect and positively indirect effect on the self-reported oral health status of MEFC, the total effect of SES on self-reported oral health status was 0.24, signified that SES had a positive effect on self-reported oral health status and in this model, the correlation coefficient between two variables was 0.24. This finding was similar to the previous study in Sarajevo which was found the positive effect between SES and self-reported oral health status was also found among children [62]. Specially, it was found that education level was positively influenced the self-reported oral health status, which was similar to the study in South-East Norway [41]. It was also found that income was positively influenced the self-reported oral health status and was similar to the study among Pregnant Women [63]. Finally, it was found in this study that job before retire was also positively associated with self-reported oral health status, which was similar to the previous study [15].

#### Relationship between social support and self-reported oral health status

Positive association between social support and self-reported oral health status was found in this study, implying the higher level of social support of MEFC, the higher level of their self-reported oral health status. The effect of social support on self-reported oral health status was 0.17, signified that social support had a positive effect on self-reported oral health status and in this model, the correlation coefficient between two variables was 0.17. Previous study had shown that social support had a significant effect on health among the old adults [64], which was similar to the findings of this study. Moreover, the financial support was found to be positively associated with the oral health [65]. Due to the social support of MEFC generally tends to be higher when they live with their children, and the health-related knowledge received from their children after living together during daily communication would also improve the self-reported oral health status of the MEFC to a certain extent.

#### The mediating effect of social support

The relationship between SES and self-reported oral health status was partially mediated by social support with an effect size of 12% and the SES indirect mediating effect via social support was 0.03, signified that social support had a positive mediating effect between SES and self-reported oral health status and in this model, the correlation coefficient was 0.03. Previous studies had already found that the social support was positively associated with oral health [27, 64], indicating the higher level of social support, the more likely to have good self-reported oral health status. A study had shown that SES could promote the health status by increasing social support among Chinese older adults [64]. These findings were similar to the results of this study, that is, the higher SES, the more social support, the better oral health of the elderly [66]. This study showed that SES and social support had positive effects on self-reported oral health status of MEFC, and SES had an indirect positive effect on self-reported oral health status of MEFC through social support. MEFC with higher SES could generally have more resources and social support, which would furtherly have a positive impact on their self-reported oral health status.

### Implications

Based on the results above in this study, targeted suggestions on improvement of SES, social support and promotion of the oral health of the MEFC were given as follow.

Firstly, family members, especially the children of MEFC, should provide more social support (both financial and emotional support) for the MEFC, and bring them more knowledge about oral health care while providing social support, so that the MEFC could understand the scientific methods of protecting the oral cavity. Secondly, the community could design more programs and create activities for the MEFC, which could increase the interpersonal communication between the MEFC and their neighbors and friends, enhance the social support level of MEFC. Thirdly, the primary healthcare center could conduct health education activities in conjunction with various health themes and distributes oral health-related educational materials for MEFC to promote health behaviors and improve their health through health education. Finally, government could increase the pension fee to increase the household income; concerning the educational attainment, lifelong learning policy should be established and promoted among MEFC by the government.

### Limitations

There are several limitations to this study. Firstly, because of the COVID-19 pandemic, we only completed the questionnaire survey in Weifang and failed to start the survey in Shanghai as planned, which may provide more information about MEFC in China. Secondly, according to Baron and Kenny’s study [67], the independent variable, dependent variable and mediation variable are all required to be continuous variables for the classic mediation effect analysis, while the independent variables (SES) were categorical variables in the current research, this may influence the result of this study. Thirdly, previous studies also clarified the effect of self-efficacy [68], gender [18], smoking [69] on oral health yet were not shown in the current study, thus more researches are needed in the future to explore these variables’ (such as confounding bias) effect on the association between SES and self-reported oral health status among MEFC.

## Conclusion

In summary, to the best of our knowledge, this study is the first to explore the mediating effect of social support between SES and self-reported oral health status among the MEFC in Weifang, China. The main findings of this study were: (1) higher GOHAI score of MEFC indicated their better self-reported oral health status; (2) MEFCs’ SES exerted positive effect both on self-reported oral health status and social support, and (3) the mediating effect of social support between SES and self-reported oral health status of MEFC was established. Targeted implication for the government, primary healthcare center, community and family members were proposed to improve the self-reported oral health status of MEFC.

## Data Availability

Under reasonable requirements, the data and material of this study can be obtained from the corresponding author. The data are not publicly available due to privacy restrictions.

## Abbreviations

MEFC: Migrant elderly following children
SES: Socioeconomic status
SSRS: Social Support Rating Scale
GOHAI: Geriatric Oral Health Assessment Index scale
SEM: Structural equation modeling
CI: Confidence interval
PSUs: Primary sampling units
SSUs: Secondary sampling units
RMB: Renminb
S.D.: Standard deviation
CMIN: Chi square
DF: Degree of freedom
GFI: Goodness of Fit Index
AGFI: Adjusted Goodness of Fit Index
NFI: Normed Fit Index
IFI: Incremental Fit Index
CFI: Comparative Fitness Index
RMSEA: Root-mean square error of approximation
